# Evaluation of antimicrobial activity of sutures soaked in injectable platelet rich fibrin or platelet rich fibrin lysate against common oral pathogens: A pilot study

**DOI:** 10.34172/japid.025.2240

**Published:** 2025-09-15

**Authors:** Megha Hulumanderi Ravikumar, Raison Thomas, Rucha Shah, Chandan Shivamallu, Chandan Dharmashekhar, Triveni Mavinakote Gowda

**Affiliations:** ^1^Department of Periodontology, Bapuji Dental College and Hospital, Davangere, India; ^2^Department of Biotechnology & Bioinformatics, Jagadguru Sri Shivaratreeshwara Academy of Higher Education & Research, Mysuru, India; ^3^Department of Life Sciences, JSS Medical College and Hospital, Mysuru, India

**Keywords:** Antibacterial, Platelet-rich fibrin, Surgical wound infection, Suture

## Abstract

**Background.:**

Suture materials in the oral cavity can promote bacterial adhesion and contamination. While antimicrobial-coated sutures are effective, their use is limited by cost, availability, and toxicity. Platelet-rich fibrin (PRF) derivatives have shown antimicrobial activity against periodontal pathogens. This study compared the antimicrobial efficacy of sutures soaked in saline, i-PRF, and PRF lysate.

**Methods.:**

An in vitro study was used to determine the minimum inhibitory concentration (MIC) of sutures soaked in saline, i-PRF, and PRF lysate. The sutures were tested against *Streptococcus mutans*, *Prevotella intermedia*, and *Porphyromonas gingivalis* by measuring inhibition zones. Non-absorbable 3-0 black silk sutures were cut and soaked for 10 minutes in saline (group A), i-PRF (group B), or PRF lysate (group C), then incubated anaerobically at 37 °C for 24 hours. Additionally, samples from all three groups were incubated at 37 °C in 5 mL of saliva from patients with chronic periodontitis, and colony-forming units (CFUs) were assessed on days 1, 3, 5, and 7.

**Results.:**

Sutures soaked in i-PRF and PRF lysate demonstrated a statistically significant increase in the zone of inhibition and a reduction in CFU against *S. mutans*, *P. intermedia*, and *P. gingivalis*. Scanning electron microscopy analyses showed a fibrin network on sutures soaked in i-PRF and PRF lysate.

**Conclusion.:**

The antimicrobial efficacy of i-PRF- and PRF lysate-treated sutures against oral pathogens appears promising. These biologically enhanced sutures may serve as effective alternatives to conventional antimicrobial sutures.

## Introduction

 Chronic periodontitis is a prevalent inflammatory condition that significantly affects oral health globally. Studies estimate that 10%–15% of the global population experiences its advanced stages.^1^ Nonsurgical periodontal therapy effectively manages early inflammation; surgical approaches such as open flap debridement are preferred for deep pockets due to their superior clinical outcomes.^2^

 Optimal healing after surgery depends on minimising microbial infiltration and achieving primary wound closure. Sutures play a crucial role in maintaining tissue approximation, promoting hemostasis, and facilitating optimal wound healing. However, they may also serve as a medium for bacterial adhesion and proliferation, increasing the risk of postoperative infection.^3,4^ Factors such as suture composition, filament structure, and interaction with oral fluids contribute to bacterial colonization. Braided sutures, like silk, are widely used due to their tensile strength and handling characteristics; however, they have been shown to allow microbial ingress via capillary wicking.^5^

 To reduce microbial adhesion, various antimicrobial-coated sutures have been introduced. Agents such as chlorhexidine and triclosan have demonstrated bacteriostatic effects, with triclosan also exhibiting anti-inflammatory benefits.^6^ However, questions persist regarding their long-term safety and efficacy, particularly concerning microbial resistance.^7^

 Platelet-rich fibrin (PRF), an autologous concentrate rich in bioactive molecules, has gained popularity in regenerative dentistry. Injectable PRF (i-PRF) and PRF lysate, derived through differential centrifugation and compression, are rich in growth factors and antimicrobial peptides.^8^ These have been investigated for enhancing wound healing, but limited evidence exists regarding their role in reducing microbial adhesion to suture materials. Therefore, this pilot study aimed to evaluate and compare the antimicrobial efficacy of sutures treated with saline, i-PRF, and PRF lysate, with a focus on their potential to inhibit bacterial colonization after surgery.

## Methods

 Ethical clearance was obtained (BDC/Exam/548/2021-2022), and the study adhered to the principles outlined in the Declaration of Helsinki. An in vitro experimental design was used to evaluate the antibacterial properties of sutures coated with saline (group A), i-PRF (group B), and PRF lysate (group C). Healthy male volunteers aged 18‒24 years were selected from theoutpatient periodontics department. After obtaining informed consent, subjects meeting the inclusion criteria were enrolled. Exclusion criteria included individuals with compromised medical conditions, smokers, alcohol users, tobacco chewers, or those on anticoagulants or bisphosphonates.

###  Sample Size

 Sample size for this study was estimated at n = 30, i.e., n = 10 in each group, according to the protocol outlined by Whitehead et.al. for a pilot trial.^9^

###  Preparation of i -PRF and PRF Lysate

 i-PRF: 9 mL of venous blood was collected into a sterile green-capped S-PRF tube (Choukron S-PRF, Process for PRF, Nice, France), followed by centrifugation at 700 rpm for 3 minutes (PRF Duo Quattro, Nice, France).^10^

 PRF lysate: 9 mL of venous blood was collected into a sterile red-capped A-PRF tube (Choukron A-PRF, NICE, France) and centrifuged at 1300 rpm for 8 minutes. The clot was compressed to form a membrane, and the collected exudate was used as PRF lysate (Process for PRF, Nice, France).^11^

###  Preparation of Suture Material

**Figure 1:**
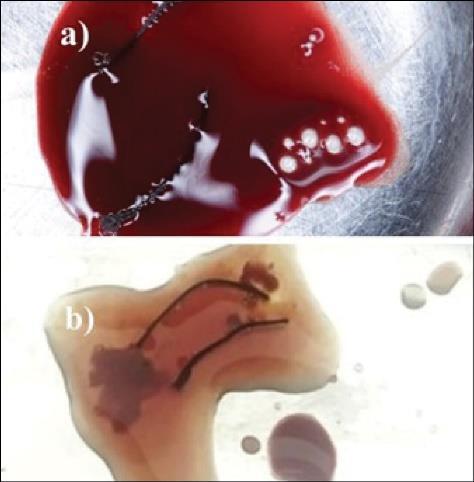
Sutures soaked in (a) i-PRF and (b) PRF lysate

 Segments of 3-0 non-absorbable black silk sutures (Lifeline, Bengaluru, India) were soaked in 2 mL of saline, i-PRF, or PRF lysate for 10 minutes (Figure 1).

###  Microbiological Analysis: Zone of Inhibition and Inoculum Preparation

 The antibacterial effectiveness of sutures was tested against *S. mutans* (ATCC 25175), *P. intermedia* (ATCC 25611), *P. gingivalis* (ATCC 33277), *F. nucleatum* (ATCC 25586), and *A. actinomycetemcomitans* (ATCC 43718). Inocula were prepared, and turbidity was adjusted to an 0.5 McFarland standard. Sterile swabs were used to evenly inoculate agar plates, which were allowed to rest for 3–15 minutes before the wells were created.

 Suture samples were placed on inoculated agar plates for 10 minutes, followed by anaerobic incubation at 37 °C for 24 hours. The zones of inhibition were measured in millimeters using a digital Vernier caliper (Insize Digital Calipers, India), and all the tests were performed in triplicate.

###  Total Colony-Forming Units (CFU)

 Sutures were incubated at 37 °C in 10 mL of saliva from patients with chronic periodontitis for 1, 3, 5, and 7 days. After agitation in saline and dilution, 0.1 mL was plated on Mueller-Hinton agar. Bacterial colonies were counted after 48 hours and reported as CFU/mL.

###  Scanning Electron Microscopy (SEM)

 Sutures were fixed in 0.25% glutaraldehyde for 48 hours at 4 °C, then dehydrated through a series of ethanol concentrations (30‒100%), and incubated for 10 minutes at each concentration, except for 100% ethanol, which was incubated for 1 hour. The samples were mounted and analyzed under a Zeiss EVO LS 15 SEM at × 100 to × 5000 magnification. (Zeiss EVO LS 15, Zeiss Microscopy, Germany).

###  Statistical Analysis

 Two-way ANOVA was performed using GraphPad software version 8 (Dotmatics, Boston). A *P* value of < 0.05 was considered statistically significant. The zone of inhibition was analyzed using Tukey multiple comparison tests, while CFU counts were analyzed using Dennett’s or Sidak’s multiple comparison tests, depending on the comparison group. The results were presented as mean ± SEM (n = 3).

## Results

**Figure 2:**
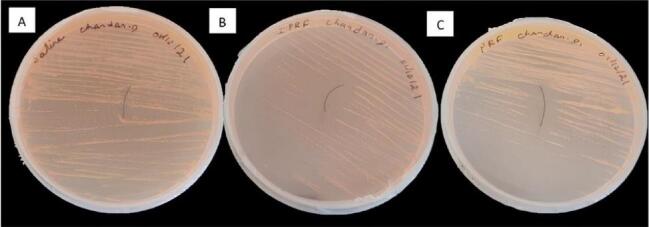
Zone of inhibition (in mm) indicated around sutures soaked in (A) saline, (B) i-PRF, and (C) PRF lysate against *Porphyromonas gingivalis* (ATCC No: 33277) in Mueller-Hinton agar

**Figure 3:**
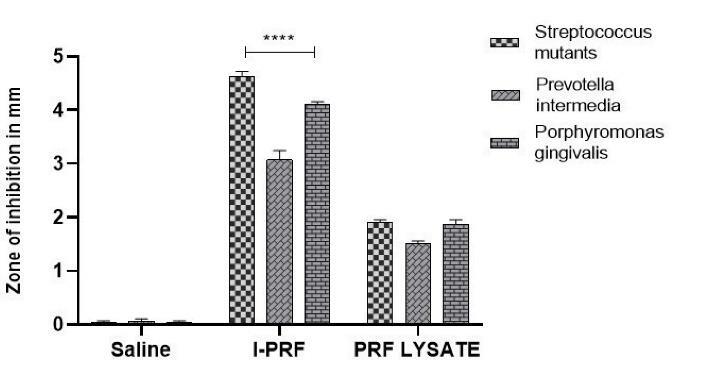
Graphic representation of comparison of the mean zone of inhibition around sutures soaked in saline, i-PRF, and PRF lysate

**Figure 4:**
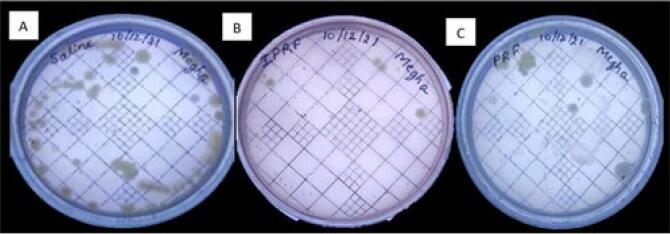
Colony-forming units obtained using sutures soaked in (A) saline, (B) i-PRF, and (C) PRF lysate

**Figure 5:**
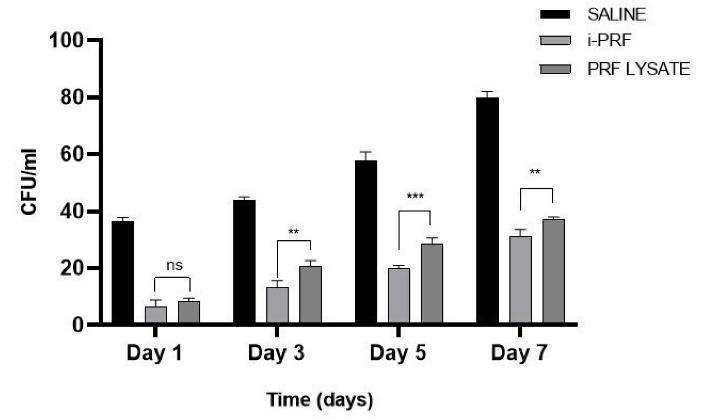
Graphic representation of total colony-forming units obtained by suspension of sutures soaked in saline, i-PRF, and PRF lysate in a patient with chronic periodontitis

 The comparison of the mean zone of inhibition around sutures soaked in saline, i-PRF, and PRF lysate is shown in Table 1 (Figures 2 and 3). Similarly, the comparison of the mean total CFUs obtained from sutures soaked in saline, i-PRF, and PRF lysate is provided in Table 2 (Figures 4 and 5).

**Figure 6:**
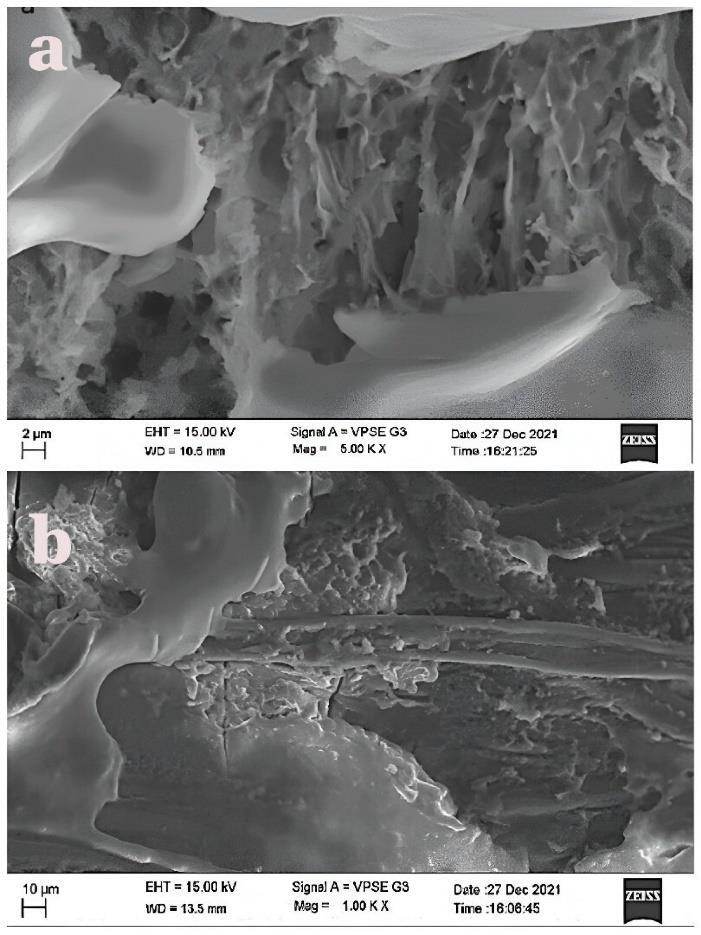
Scanning electron microscopy images of sutures dipped in (a) i-PRF and (b ) PRF lysate

 SEM analysis of sutures soaked in i-PRF revealed the fibrin network structure and cellular components on the surface of 3-0 black silk sutures. The fibrin network was dense, with cells visible at higher magnifications. In contrast, SEM analysis of sutures soaked in PRF lysate showed a minimal number of cells and a loosely connected fibrin network (Figure 6).

**Table 1:** Comparison of the mean zone of inhibition present around sutures soaked in saline, i-PRF, and PRF lysate

**Group comparison**	**Pathogenic bacteria**	**Mean±SEM**	***P *** **value**
Saline vs. i-PRF	*Streptococcus mutants*	-4.600 ± 0.090	< 0.0001**
	*Prevotella intermedia*	-4.600 ± 0.090	< 0.0001**
	*Porphyromonas gingivalis*	-4.067 ± 0.0902	< 0.0001**
Saline vs. PRF lysate	*Streptococcus mutants*	-1.867 ± 0.090	< 0.0001**
	*Prevotella intermedia*	-1.433 ± 0.154	0.0002**
	*Porphyromonas gingivalis*	-1.833 ± 0.0902	< 0.0001**
i-PRF vs. PRF lysate	*Streptococcus mutants*	2.733 ± 0.1155	< 0.0001**
	*Prevotella intermedia*	1.567 ± 0.1155	< 0.0001**
	*Porphyromonas gingivalis*	2.233 ± 0.1155	< 0.0001**

*P < 0.05 statistically significant, **P < 0.001 statistically highly significant, Two-way ANOVA with Tukey’s multiple comparison test.

**Table 2:** Comparison of the mean of total colony-forming units obtained by suspension of sutures soaked in a) saline, b) i-PRF, and c) PRF Lysate in a chronic periodontitis patient

**Days**	**Group comparison**	**Mean±SEM**	***P*** ** value**
Day 1	Saline vs. i-PRF	30.00 ± 1.700	0.0004**
	Saline vs. PRF lysate	28.00 ± 1.106	< 0.0001**
	i-PRF vs. PRF lysate	-2.000 ± 1.667	0.6795
Day 3	Saline vs. i-PRF	31.00 ± 1.633	0.0013*
	Saline vs. PRF lysate	23.33 ± 1.333	0.0008**
	i-PRF vs. PRF lysate	-7.667 ± 1.667	0.0012*
Day 5	Saline vs. i-PRF	37.67 ± 1.944	0.0018*
	Saline vs. PRF lysate	29.33 ± 2.285	0.0006**
	i-PRF vs. PRF lysate	-8.333 ± 1.667	0.0005**
Day 7	Saline vs. i-PRF	49.00 ± 1.915	< 0.0001**
	Saline vs. PRF lysate	43.00 ± 1.291	0.0001**
	i-PRF vs. PRF lysate	-6.000 ± 1.667	0.0096*

*P < 0.05 statistically significant, **P < 0.001 statistically highly significant, Two-way ANOVA Sidak’s multiple comparisons test.

## Discussion

 Bacterial colonization of sutures is a significant risk factor for infections, bacteremia, and endocarditis following dentoalveolar surgeries, as sutures in the oral cavity can facilitate bacterial adhesion and wound contamination.^4^ A clean, bacteria-free environment at the surgical site is vital for recovery, and systemic antibiotics are commonly used to ward off postoperative infections. However, in immunocompromised patients, antibiotics are often essential but present drawbacks, such as antibiotic resistance and inadequate local drug concentrations that fail to achieve the minimum inhibitory levels needed to control pathogens in the oral cavity.By delivering sustained antimicrobial effects locally, antibacterial-coated sutures provide an effective means of infection control, potentially reducing the need for systemic antibiotics. Currently, chlorhexidine- and triclosan-coated sutures are commercially available. Studies have demonstrated their effectiveness in reducing bacterial colonization on sutures and preventing surgical site infections.^5^ However, their usage is limited by factors such as low availability, high cost, and potential side effects.^7^

 Platelet concentrates such as PRF offer dual benefits—tissue regeneration and antimicrobial action. i-PRF, prepared through low-speed centrifugation, retains leukocytes, platelets, and growth factors, thereby enhancing its antibacterial capabilities through the release of cytokines, peptides, and enzymes.^12,13^ Multiple investigations have assessed the antimicrobial properties of platelet concentrates. A systematic review by Balaji et al^14^ evaluated the antimicrobial efficacy of various PRF types in clinical and in vitro studies. Eight studies were included, demonstrating the effectiveness of PRF against pathogens by inhibiting bacterial growth. The enhanced effect may be linked to platelet release and preparation methods. Kour et al^15^ found that i-PRF and PRP had stronger antibacterial activity than PRF against *P. gingivalis* and *A. actinomycetemcomitan*s. With its simple preparation process and biocompatible nature, i-PRF is a valuable tool in surgical therapy, aiding in both tissue regeneration and bacterial control.

 Derived via a low-speed centrifugation protocol, i-PRF retains a high concentration of platelets and white blood cells. These elements support its slow-release capacity for antimicrobial peptides and growth factors such as HBD-3 and myeloperoxidase, enabling both regenerative and antibacterial effects at the site of application.^16^

 In the current investigation, sutures immersed in i-PRF exhibited more potent antimicrobial activity on days 1 and 3, with a noticeable decline by days 5 and 7, as demonstrated by the zones of inhibition against *S. mutans*, *P. gingivalis*, and *P. intermedia*. This pattern of initial potency followed by a progressive reduction is consistent with observations by Ravi et al,^17^ who reported PRF degradation over seven days under chemical stress. These results are also consistent with a study by Kour et al,^15^ which demonstrated superior antimicrobial activity of i-PRF compared to PRP and PRF against periopathogens.

 The lysate obtained from PRF membranes, created by compressing PRF clots, includes angiogenic^6^ mediators such as cytokines and structured glycoproteins, which may accelerate early periodontal wound healing.^18^ Sutures treated with PRF lysate demonstrated antimicrobial activity against *P. gingivalis, S. mutans*, and *P. intermedia*, showing clear inhibition zones and reduced CFU counts on days 1 and 3. This effect is attributed to hydrogen peroxide- and peptide-mediated bacterial lysis.^19^ However, the inhibition zones and CFU suppression were less pronounced compared to i-PRF, though more effective than saline.

 The potent antimicrobial effect of i-PRF arises from proteins such as defensins, cathelicidins, lactoferrin, and phospholipase A2, which disrupt bacterial functions and lead to cell death.^20^ In addition, its cellular components—leukocytes and platelets—boost antimicrobial peptide production, further strengthening its antibacterial action.

 Autologous platelet concentrates such as i-PRF and PRF lysate may serve as effective adjuncts for infection control, particularly in patients with systemic conditions like diabetes mellitus, where impaired wound healing and a heightened risk of surgical site infections are prevalent. In regenerative periodontal therapy, PRF lysate obtained during membrane preparation can be repurposed to soak suture materials, thereby enhancing their antimicrobial properties.

 This study, however, has some limitations. The antimicrobial activity may vary depending on the physical structure and absorption capacity of different suture types. Furthermore, the longevity of the antimicrobial effect of sutures treated with i-PRF and PRF lysate remains to be evaluated. Further in vivo studies are essential to validate these preliminary findings and confirm the clinical potential of this approach.

## Conclusion

 This study concludes that sutures soaked in i-PRF and PRF lysate demonstrated significant antimicrobial activity against common oral pathogens. Among these, i-PRF showed the highest efficacy. These sutures may serve as a viable alternative to commercially available antimicrobial-coated sutures. Further research is needed to confirm these findings.

## Competing Interests

 The authors of the current manuscript declare no conflicts of interest regarding the publication of the presented paper.

## Data Availability

 Available on request.

## Ethical Approval

 Obtained from the institutional review board and ethics committee (BDC/Exam/548/2021-2022).
